# Operationalization of One Health Burnout Prevention and Recovery: Participatory Action Research-Design of Nature-Based Health Promotion Interventions for Employees

**DOI:** 10.3389/fpubh.2021.720761

**Published:** 2021-09-07

**Authors:** Ann Sterckx, Kris Van den Broeck, Roy Remmen, Kathleen Dekeirel, Hildegard Hermans, Carmen Hesters, Tine Daeseleire, Viki Broes, Jo Barton, Valerie Gladwell, Sarah Dandy, Michéal Connors, Annamaria Lammel, Hans Keune

**Affiliations:** ^1^Chair Care and the Natural Living Environment, Department of Primary and Interdisciplinary Care, Faculty of Medicine and Health Sciences, University of Antwerp, Antwerp, Belgium; ^2^Chair Public Mental Health, Department of Primary and Interdisciplinary Care, Faculty of Medicine and Health Sciences, University of Antwerp, Antwerp, Belgium; ^3^Department of Primary and Interdisciplinary Care, Faculty of Medicine and Health Sciences, University of Antwerp, Antwerp, Belgium; ^4^Human Resources Department, Antwerp University Hospital, Edegem, Belgium; ^5^Department Human, Communication, Organization, Antwerp, Belgium; ^6^The Human Link, Let's Talk About Mental Health, Antwerp, Belgium; ^7^Vereniging van Erkende Stress en Burn-out Coaches, Brussels, Belgium; ^8^Vereniging Zonder Winstoogmerk Burnout Vlaanderen, Berlaar, Belgium; ^9^School of Sport, Rehabilitation and Exercise Sciences, University of Essex, Colchester, United Kingdom; ^10^The Centre for Sustainable Healthcare Cranbrook House, Oxford, United Kingdom; ^11^The Natural Academy, Bristol, United Kingdom; ^12^Department Psychology, Laboratory Paragraphe, University Paris-8, Saint-Denis, France

**Keywords:** burnout, one health, nature-based, health promotion intervention, participative action research, mental health, nature connectedness, biophilic design

## Abstract

Burnout is, besides a global, complex phenomenon, a public health issue with negative consequences on personal, organizational, social, and economic levels. This paper outlines the co-design of a novel Nature-based Burnout Coaching intervention, called NABUCO. Due to the complexity of burnout, we propose a One Health approach in healthcare, educational and governmental pilot organizations, to deliver guidelines and protocols for prevention and recovery of burnout. We advocate the inclusion of the salutogenic and mutual healing capacity of nature connectedness, facilitating a positive impact on mental and environmental health. A transdisciplinary Participative Action Research-design resulted in an iterative adaptive cycle of co-design, implementation, and evaluation of NABUCO.

## Introduction

Burnout is a silent crisis imposing significant costs on individual's health and the wider global economy. Urgent action is required to identify effective complementary interventions for prevention and recovery of burnout. In this paper, we demonstrate how to operationalize a One Health approach (OH-approach) to burnout interventions. To start, we discuss the complexity of burnout and the relevance of a OH-approach. Next, we describe why the inclusion of the mutual healing capacity of nature connectedness should be included in the burnout intervention.

The World Health Organization defined burnout in the 11th Revision of the International Classification of Diseases (ICD-11) as an occupational phenomenon (1). Burnout is, besides a global complex phenomenon (2–4), also a public health crisis (5) with negative consequences on individual, organizational, societal and economic factors, appearing in sectors such as healthcare (6–8), education (9), and government (10). Due to international differences in the use of the term burnout (11), for this research burnout is defined as “a work-related syndrome involving emotional exhaustion, depersonalization, and a sense of reduced personal accomplishment” (12–14).

Burnout interventions should be considered within a model of health promotion interventions (HPI), considering the structural, social, and cognitive complexity (15). Each kind of complexity and its mutual interactions present challenges for the prevention and recovery of burnout. For instance, structural complexity arises as several players are involved at different steps in the process of HPI, ranging from the outset of the employee's burnout to the re-integration phase at work. Next, cognitive complexity is found in the emergence of complex decision processes due to the high number of interrelationships and interdependent decisions between these players. Consequently, accurate outcomes of the HPI are hard to predict. Finally, the variety of the relationships involved within and between the individual, organizational and societal contexts, can give rise to disagreement or social conflict, also called social complexity. Furthermore, assuming burnout is not solely job-related [e.g., parental burnout (16)], Bianchi (17) proposes to perceive burnout as a “*multi-domain syndrome.”* Nonetheless, organizations often apply person-directed HPI (18) (e.g., counseling, mindfulness exercises), suggesting burnout is an isolated problem to be solved by the individual (19) and is limited to the context of work. Consequently, attention to the whole system and larger settings, in which employees suffering from burnout reside, is often lacking (20). Therefore, choosing a OH-approach in tackling the problem of burnout could be more appropriate. A OH-approach consists of “a collaborative, multisectoral, and transdisciplinary approach—working at local, regional, national, and global levels—with the goal of achieving optimal health outcomes, recognizing the interconnection between people, animals, plants, and their shared environment” (21, 22). The OH-approach promotes a holistic, integrative, and transdisciplinary perspective to address complex health threats (21). In short, a holistic health approach assimilates the relationship between mind, body, and emotion within the person (23), situated within a broader context, in which human, social, and environmental health determinants are intertwined (24). For instance, a person's health and quality of life is not merely influenced by their work environment but also by lifestyle changes, and the social, economic, and natural environments (25–27) in which the person is situated. Furthermore, an integrative perspective on burnout should be incorporated in the HPI. For instance, a combination of person- and organizational-directed interventions offers the potential for more effective rehabilitation (18). Finally, applying a transdisciplinary perspective, which is well-known in HPI, sustainability science (23, 28, 29) and the OH-approach (21, 22), may “produce highly novel and generative scientific outcomes” (28). While integrating voices of all the stakeholders in the design and deployment of the HPI, a transdisciplinary approach entails bridging science, professional expertise and other sectors.

Alongside this, the salutogenic and mutual healing capacity of nature connectedness (NC) could be a promising mediator in the prevention and recovery of burnout, while at the same time contributing to environmental health. There is a growing body of evidence of the salutogenic effects of contact with nature, with positive psychological (30–34), cognitive (35–37), physiological (31, 38–41), and social benefits (42, 43). Although the translation of this knowledge into health practice is not common (44), “ecotherapy” is becoming a germinating field in healthcare (45, 46). Ecotherapists are mainly mental health professionals, additionally trained in guiding clients with Nature-based Interventions (NBI). For this study, NBI is defined as “planned, intentional activities to promote individuals” optimal functioning, health and well-being or to enable restoration and recovery through exposure to or interaction with e.g., either immersive or authentic nature (47). NBIs encourage employees to engage with nature, to receive multiple health benefits on several levels (e.g., behavioral and lifestyle change and changes in the work environment) (47). Although there is heterogeneity in scientific evidence, positive effects on mental health, cognitive ability, recovery and restoration, and on life and work satisfaction, have been reported (48, 49).

Besides offering nature exposure for health purposes (e.g., general well-being, attention restoration, stress reduction), some ecotherapists focus on improving NC with their clients. NC is defined here as “…a stable state of consciousness comprising symbiotic cognitive, affective and experiential traits that reflect, through consistent attitudes and behaviors, a sustained awareness of the interrelatedness between one's self and the rest of nature.” (50) As such, NC is considered a mediator for developing a mutual relationship between the client's wellbeing and better self-care, as well as care for the natural environment (45, 50–52). For example, as a result a person might adopt a more ecological lifestyle contributing to one's health and environmental health (e.g., organic food, ecological way of transportation, introducing biodiversity in the garden). This “active two-way nurturing of human and nature” (51), evoked by NC, supports the mutual healing for the person and the natural environment. Recent research shows that NC, besides being a psychological need (53), positively influences the quality of life, brings meaningfulness, happiness, and vitality (35, 50, 54, 55). Furthermore, NC fosters pro-environmental behavior (56) and stimulates a holistic reciprocal relationship with nature (57). Besides including NC within a personal context, incorporating natural elements into the workplace can also be beneficial (58–61). A notable example is biophilic design (62), which aims to create restorative environments and improve people's NC (62–64) contributing on an organizational level to employee wellbeing, productivity, and mitigating stress (65, 66). Despite these promising mutual health benefits, organizations, and general practitioners appear to be cautious in adopting and prescribing NBIs. Besides the lack of resources and time (42), the absence of a proven professional and evidence-based framework, and of natural spaces nearby the organization or the employee's home, could be behind this hesitation.

In conclusion, the NBI operationalizing a OH-approach, while integrating the focus on improving NC, may lead to several mental health benefits, a sustainable individual behavioral lifestyle change, and changes on organizational level. These may affect employee's, organizational and environmental health. Developing a professional and evidence-based framework in close collaboration with the stakeholders, while using local health and environmental knowledge (22, 51, 67), can lead to NBI protocols and guidelines for healthcare professionals and organizations. As a result, confidence and leverage might increase in choosing NBIs as a complementary approach to the prevention and recovery of burnout.

## Aim and Objectives

**Figure 1:**
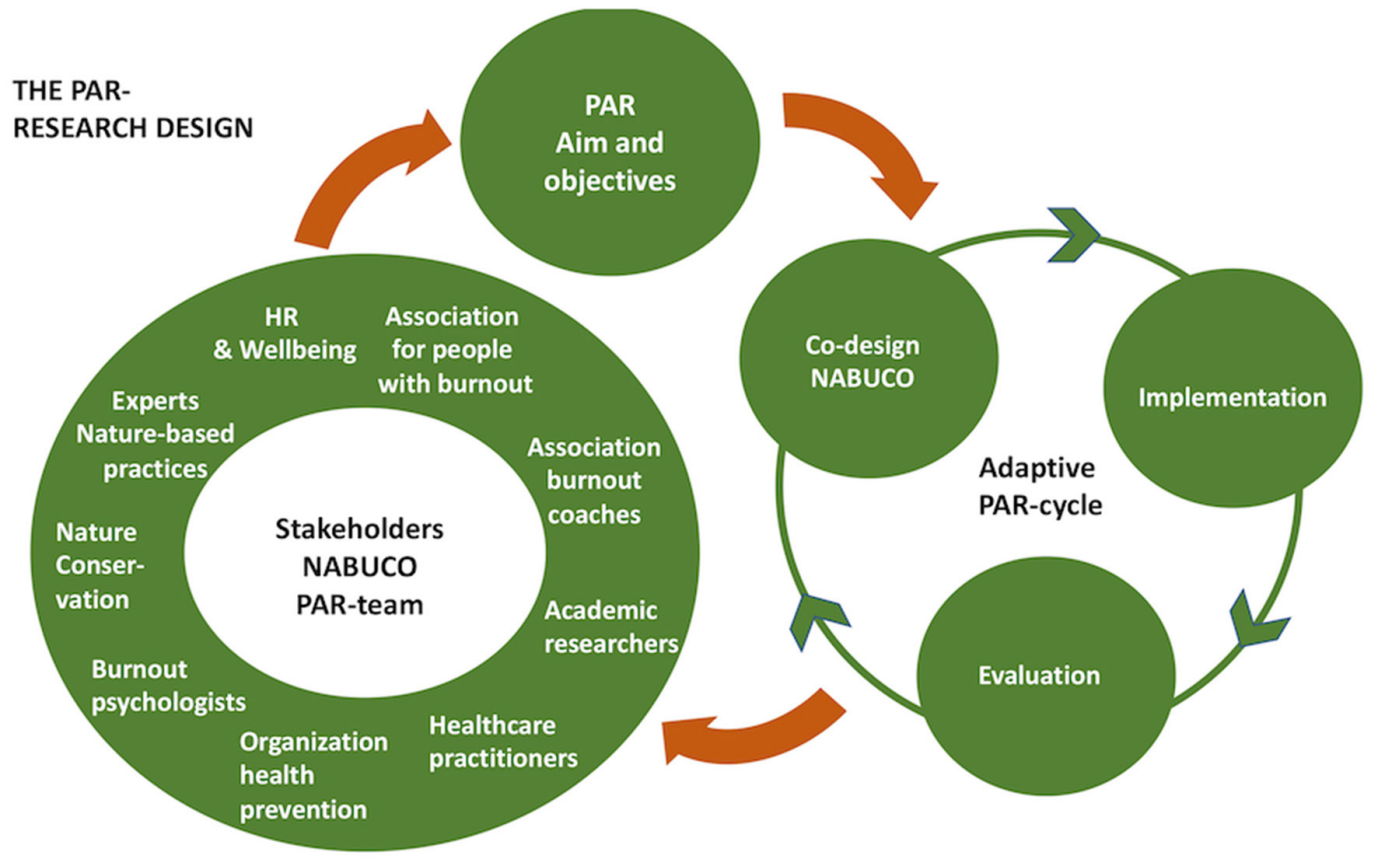
The PAR Research design. The adaptive PAR-cycle encompasses cyclical information exchange. In a collaborative process, the aim, the objectives and the NABUCO study were refined.

This paper reports the formative co-design process of NABUCO, using the principles of the Participative Action Research (PAR) design (Figure 1), and how we are operationalizing a OH-approach to burnout. Piloting, implementation and evaluation of NABUCO will be the subject of a sequential project following this formative co-design stage. A systematic review of NBIs will be conducted as well.

Based upon conversations with key stakeholders of NABUCO, this paper will explore the following research questions:

1. How to co-design NABUCO in an organizational context within an evaluative framework.
2. How a OH-approach can tackle the complexity of burnout and work toward sustainable results on a personal, organizational, societal, and environmental level.
3. How NABUCO can facilitate a positive impact on prevention and recovery from burnout within an organizational context.
4. How and what we can learn from the transdisciplinary co-design process of this complex HPI.

## Methods

In this section we discuss the research design, its setting, the participants and the co-design process.

### The Research Design: Participative Action Research (PAR)

We chose a Participatory Action Research (PAR) methodology. PAR is a research approach in which knowledge is constructed collectively through iterative cycling between phases and actions, while inducing a change in a certain context (68). It is also used to develop complex interventions (69, 70), where “participation of stakeholders from definition to resolution” (22) is crucial. For ethical reasons and to create leverage for NABUCO, involving those impacted the most by the problem (70, 71) and using local knowledge and experience (51, 67, 68) is significant, whilst building bridges between stakeholders and different disciplines. The PAR-design (Figure 1) is supported by an adaptive non-linear PAR-cycle, characterized by planned and spontaneous interplay between the phases of co-design, implementation and evaluation. A collaborative process refined the aim, objectives and the study design. We also discussed how to assess the PAR quality. Criteria such as the level of participation and collaboration between the PAR-team members, critical reflexivity, how actions are locally situated, and different kinds of validity to evaluate within PAR (68), will be discussed in depth at next PAR-rounds.

### Setting and Participants

We initially explored the topic with experts in burnout and nature-based practices. Next, we assembled a transdisciplinary group of key stakeholders, later referred to as the PAR-team, from different domains and different countries (Table 1). The stakeholders have been selected based on their interest in the project, relevant experiences and expertise.

**Table 1:** Composition of the PAR-team members by profession and country.

**Professional PAR-Team member**	**Country**
	**Belgium (BE), The Netherlands (NL), United Kingdom (UK), France (FR)**
Personnel manager	BE, NL
Well-being manager	BE
Academic researchers	BE, UK, BE, UK, FR
Representative of the Association of professional burnout coaches	BE
Health psychologist	BE
Burnout coach	BE
Spokesperson of the Association of burnout victims	BE
Representative of a nature conservation organization	BE
Training coordinator of coaching in nature	BE
Representative of an association for healthcare organizations	NL, UK
Nature-based health practitioners	BE, UK, NL

### The Co-design Process

The co-design process consists of four sub-processes: data collection, exploration and capacity building, the intervention and its evaluation.

#### Data Collection

Data collection, exploring several topics suggested by all the PAR-team members, was achieved through questionnaires, storytelling, digital and physical group conversations, observations and content notes of the facilitator. They were further supported by gray and scientific literature. The data analysis adopted different approaches according to the methods of data collection. For instance, data from conversations and group discussions led to collective interpretation and negotiation with the PAR-team members (71). In another approach, one researcher analyzed data gathered by questionnaires (for example, regarding the design of the evaluation process of NABUCO). As a result, the PAR-team verified all reported results.

#### Exploration and Capacity Building

First, the PAR-team reflected on shared motivations, values, expectations and collaboration. We also gathered local knowledge, embodying the stakeholders' perceived problems and opportunities regarding burnout prevention and recovery, the different contexts and countries in which they operate, and the capacity building to support a new intervention. Next, we explored the preferred NABUCO outcomes, which fed into subsequent NABUCO-protocols.

#### The NABUCO Intervention

The PAR-team co-designed the framework, content and protocols for NABUCO. An iterative process allowed for moving back and forward between data collection and interpretation. Reports and presentations of the collected data informed the PAR-team on how to elaborate on the steps, actions, and practices being considered in NABUCO. This led to the core elements of NABUCO (see Results).

#### The Evaluation of the NABUCO Intervention

Academic PAR-team members designed an online questionnaire about which factors to evaluate during the future implementation of NABUCO. Discussions concerning the results, led to consensus on four points. Firstly, NABUCO-participants, HR-managers, and the NABUCO-coaches should participate in interim evaluations at different stages of the implementation of NABUCO. Some argued for evaluation input from general practitioners or psychologists. However, this could result in ethical issues (due to clients then becoming patients) and delay the intervention. Secondly, we should evaluate across all stakeholders through a range of methodologies, concretized by a mixed methods design, generating quantitative and qualitative data. The use of questionnaires, interviews, discussion- and focus groups, collecting qualitative data of the communities of practice (see Communities of practice), would support this design. Thirdly, we ranked the evaluation topics. Burnout was ranked first, closely followed by perceived stress, well-being, NC, mental health, resilience, physical health and social connectedness. Less highly ranked were productivity and individual development. Capturing challenges, barriers, opportunities in the workplace as well as side effects of NABUCO were noted as being essential to understand the implementation processes and how a NBI might function in the workplace. An additional request was to conduct a cost/benefit analysis of NABUCO and a Health Impact Assessment, measuring the psychological, physiological, and organizational effects of NABUCO in depth. This HIA enforces the mixed methods design, which mainly focuses on measuring the quality of NABUCO.

Concerning the validation on the generalizability and feasibility of NABUCO, the PAR-team suggested, besides conducting a systematic review, to involve external focus groups with stakeholders of other organizations, within the same sectors (healthcare, educational, governmental). At a further stage, we may widen the validation to other sectors.

## Results

**Figure 2:**
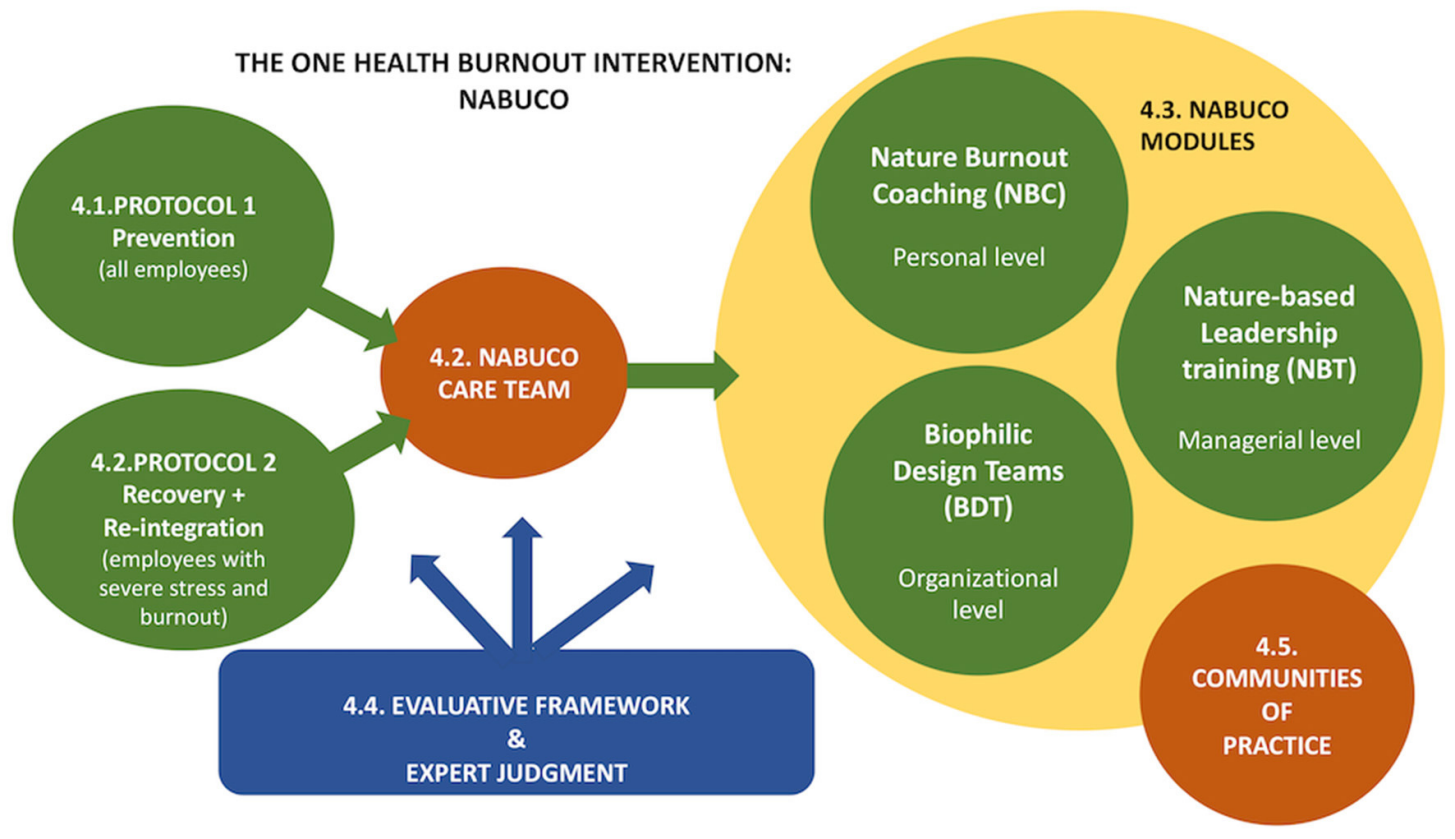
NABUCO, a nature-based One Health burnout intervention, with Protocol 1 (Prevention) and Protocol 2 (Recovery and re-integration).

The above co-design process resulted in the conception of NABUCO (Figure 2), integrating the mutual healing capacity of NC and operationalizing the OH-approach. Below we outline briefly five key elements underpinning NABUCO.

### Protocols

The PAR-team developed two NBI-protocols (P1, P2). P1 is a preventive approach for employees at risk for stress and burnout, while P2 is meant for employees at high risk of severe stress or recovery from burnout. Demonstrating the main steps to undertake in organizing the NBI, the protocols consist mainly of (1) scoping and selection of the participants in the NBI, (2) the actual participation, (3) the evaluation of the NBI and (4) the follow-up after the participation in the NBI. In P2, the re-integration phase of the employee is an additional step in the intervention.

### NABUCO-Care Team

A transdisciplinary NABUCO-care team will be assembled at the start of NABUCO within an organization. Consisting of the psychologist (responsible for expert judgment), the NABUCO-coach, and the organization's representative (e.g., HR-Department), this team will support any participant in the NABUCO-modules (see NABUCO-modules) when needed (e.g., problems between a participant and the NABUCO-coach, non-acceptance by a leader, personal issues regarding the intervention). Each team-member will be trained in NABUCO, to fully understand their role and responsibilities. Moreover, collaboration with the NABUCO-care team allows the PAR-team to adjust the research intervention when required.

### NABUCO-Modules

Some PAR-team members mentioned that NBIs would offer a complementary health approach, reaching a group of employees not attracted to the current burnout practices offered by their organization (e.g., mindfulness, CBT-therapy, indoor coaching). The PAR-team came up with a set of nature-based modules, aimed at developing sustainable individual and collective self-care capacity. They were called NABUCO-modules, consisting of nature-based burnout coaching (NBC), nature-based leadership training (NBT) and implementing biophilic design teams (BDT) within the participating organization. All modules focus on stimulating NC by offering eco-therapy exercises and profound experiences for inducing individual, organizational and environmental change. Firstly, the NBC consists of a group (in P1) or an individual (in P2) trajectory, focusing on stress-recovery and developing NC. Secondly, some mentioned the development of managers' authentic leadership as a potential mediator in the burnout prevention of employees. Considering the feasibility in the organization and acknowledging the positive role of NC in developing authentic leadership (72), an open program NBT for leaders of different participating organizations will be offered. The open program, consisting of nature-based leadership-related and NC exercises, will promote processes such as shared learning, crosspollination and mutual support between the leaders of the participating organizations. It will also foster their psychological resilience, evoking a positive effect on the employees' wellbeing and the overall organization's health. Thirdly, one of the PAR-team members mentioned the use of the natural environment in and around the workplace (e.g., walking meetings in nature, lunch break in the park, decorating the building with plants, nature wallpapers). Consequently, BDT is suggested to be implemented in the participating organizations. NBC and NBT-participants are invited to join as NABUCO-ambassadors to maintain their NC experiences and share these with their colleagues. Other interested employees are welcome to participate. As such, BDTs might be a gateway for those that suffer the most but are hard to reach. In conclusion, all NABUCO-modules will incorporate a holistic OH-approach, focusing on improving NC, mediating the reciprocal link between human, organizational and environmental health. Moreover, the layered developmental approach of the employee, the management, and the organization and the collaboration with several stakeholders in these modules brings the integrative perspective of OH into practice.

### Communities of Practice

Striving for sustainable results with NABUCO, the PAR-team proposed communities of practice aiming at (1) promoting cross-pollination of NABUCO-knowledge, experiences and best practices (2), stimulating transdisciplinary and reflexive capacity, (3) feeding the implementation of NABUCO, (4) and guaranteeing the continuation after NABUCO. Besides meeting on a regular basis physically, a virtual incubator, by means of a digital platform, gathers NABUCO-information and experiences shared by participants. These communities of practice also promote coaching and mutual support.

### Evaluation Framework and Expert Judgment

During the intervention, we will introduce expert judgment, in collaboration with an external general practitioner or psychologist, within this evaluative framework. At the start of the intervention, it will serve to categorize employee participants, in particular for those at high risk of stress and diagnosed with burnout. During the scoping phase the expert judgment examines whether these individuals are suitable for participation in the NABUCO-modules. At the end of the guidance and the re-integration, the expert evaluates the employee's progress. This evaluation framework will be developed further.

## Discussion

This paper outlines the co-design process of a complementary NBI for burnout. In the NABUCO project we consider the OH-approach, while integrating the salutogenic and mutual healing capacity of NC to be relevant and innovative. As a result, we came to a thoughtful combination of a holistic, integrative and transdisciplinary person- and organization-directed intervention, adding value to actual burnout interventions. The co-design setting creates leverage as the innovation happens from within, while staying close to the stakeholders' realities. Moreover, the data collection and collaborative interpretation created the foundation for further development of the NABUCO-implementation. However, some critical reflections and important learning points should be considered for the further development of NABUCO.

### Critical Reflections on and Lessons Learned From the Co-design Process of NABUCO

#### The Co-design Process Raised Six Critical Points

Firstly, the co-design process has built connections between stakeholders, resulting in a strong partnership, as all PAR-team members participated fully during the co-design process. They will be invited again for the continuation of this research and the further development of NABUCO. Additionally, new insights arose by sharing each other's difficulties and practices regarding tackling burnout within an organizational context. For instance, the HR-managers realized that setting up a collaboration between organizations would help facilitate mutual learning.

Secondly, in taking a transdisciplinary OH-approach in this study, the PAR-team felt the need for creating common ground by attuning each other's professional language. Deep conversations, supported by schematic visualizations and presentations avoiding the use of jargon, were vital for mutual understanding and overcoming silos.

Thirdly, a concern arose that sustaining stakeholder engagement can be challenging due to the length of the project. Literature about PAR-methodology discusses this well-known issue. Keeping the transdisciplinary aspect of the PAR-team in mind, we might need to re-confirm the members in the next co-design phase and to clarify our mutual expectations, resources, communication strategies and roles during the research (68). Nevertheless, new dynamics within the PAR-team, new content and new opportunities regarding NABUCO might arise.

Fourthly, given the ambition of full participation of all stakeholders at all phases of the PAR, involving employees considered at high risk for or even suffering from burnout in the co-design process is highly advisable. However, a PAR-member, who is also a burnout expert, mentioned that based on her experience, it would be challenging to reach those employees. For instance, stress patterns (e.g., feeling of lack of time, being overly responsible) may counter their motivation for participation. If we do not want to miss out on the purpose of this intervention, discussing the most suitable way to involve these individuals in the co-design process with them would be essential. For now, only the spokeswoman of VZW Burn-out Vlaanderen, a Flemish association of burnout victims, participated in this formative co-design phase. However, it will be imperative to integrate the voices of those who suffer the most for ethical reasons. Consequently, finding the right language and the most effective communication channels to reach these individuals (68), while ensuring a safe and ethical research setting (51, 68, 71), will be crucial in further developing NABUCO.

Fifthly, the evaluation of our collaboration and participation level is based on the facilitator's observations and comments, and on spontaneous reflective communication between the PAR-team members. Consequently, since the PAR-team has agreed on the need for a more in-depth evaluation, this will be further developed in the following co-design meeting.

Finally, we enjoyed a genuine collaborative process of reflection, learning, and action. Nevertheless, barriers concerning the co-designed protocols or our further collaboration might arise with the re-confirmed PAR-team. Time, contexts, PAR-members and resources might have changed since the formative co-design process of NABUCO. Therefore, a skilled facilitator will be important, to lead the PAR-team through these barriers, by building trust and encouraging stakeholder engagement while promoting emergent processes and quality dialogue.

### Critical Reflections on and Lessons Learned From the Content Design of the NABUCO-Intervention

This part will discuss the challenges and opportunities for the further implementation and evaluation of NABUCO put forward by the PAR-team.

With visuals and textual overviews, we thoroughly checked each NABUCO-element, regarding its content and specific contribution to the intervention, with the PAR-team. Next, feedback was gathered on the logical flow of the NABUCO-intervention as a whole, the stakeholders' role, the clarity of the steps in the process and the evaluation processes of the NBI so far.

Firstly, some PAR-members reported that implementing specific steps or actions could differ in intensity or kind due to their contexts, organizational culture, economic feasibility, and human resources. As a result, continuously adjusting the protocols and implementation processes during the PAR piloting and evaluation stages is necessary.

Second, more profound reflection is required regarding the distinct responsibilities of expert judgment. Until now, we had discussions within the PAR-team whether expert judgment should also be involved in Protocol 1 (P1). Expert judgment could be very time-consuming and cost-intensive, as the NBC in P1 targets mainly groups. As such, expert judgment should only be applied in Protocol 2, and thus be limited to individuals at high risk or diagnosed with burnout. On the other hand, a generalized expert judgment would offer objective advice on the suitability for participation in the NABUCO-modules and on the employees' progress in their self-care capacity. Both are prerequisite in developing a professional and evaluative framework. Accordingly, the topic of expert judgment will be discussed in-depth in the consecutive co-design meetings.

Third, a systematic review of NBI is essential to develop a deep theoretical understanding of “the changes and the causal chain the NBI can provoke at personal and organizational levels” (48). Moreover, concepts such as burnout and NC should be studied further in-depth to decide how to measure the impact of the NBI on all levels.

Fourth, it is difficult to distinguish between mental health issues and burnout as they may interact. NABUCO might also have a positive impact on, for example, other mental health issues or on other domains of burnout. Consequently, NABUCO could also be considered as “inclusive” and as a complementary approach, reaching those who still are at work. It will be imperative to execute a rigorous evaluation program, to gain a better insight into this issue, which will be done at a subsequent stage in our research.

Finally, the Health Impact Assessment results will support the development of a professional evaluative framework, thereby increasing the confidence of healthcare practitioners in prescribing NBI as a complementary HPI. The same is true for evaluating economic feasibility and assessing the socio-economic impact of NABUCO, which could help shape Ministry of Health policies and promote NBI prescriptions for the prevention and recovery of burnout.

## Conclusion

Despite the shortcomings and the challenges mentioned above, the co-design process has led to an innovative NBI for burnout prevention and recovery in organizations. Co-design and transdisciplinarity, operationalizing a OH-approach in the NABUCO-intervention, are vital when dealing with the complex challenges of burnout. Furthermore, mediating the reciprocal relationship with nature by improving nature connectedness for better self-care and care for the natural environment may lead to a sustainable impact at an individual, organizational and environmental level. Consequently, NABUCO could be seen as a potential complementary approach to tackle burnout in the workspace.

## Data Availability Statement

The raw data supporting the conclusions of this article will be made available by the authors, without undue reservation.

## Ethics Statement

The studies involving human participants were reviewed and approved by the committee of medical ethics, University Hospital Antwerp/University of Antwerp. The participants provided their written informed consent to participate in this study.

## Author Contributions

HK and AS had the lead in the co-design of NABUCO. AS was responsible for the different drafts of this paper. All authors have read and approved the final version of the manuscript and contributed equally to the co-design of NABUCO.

## Funding

The University of Antwerp, Chair Care and the Natural Living Environment, which was funded by the Province of Antwerp, supported this work. The Province of Antwerp funded their Personnel Department for its contribution to this research project.

## Conflict of Interest

The authors declare that the research was conducted in the absence of any commercial or financial relationships that could be construed as a potential conflict of interest.

## Publisher's Note

All claims expressed in this article are solely those of the authors and do not necessarily represent those of their affiliated organizations, or those of the publisher, the editors and the reviewers. Any product that may be evaluated in this article, or claim that may be made by its manufacturer, is not guaranteed or endorsed by the publisher.

## Abbreviations

HPI: Health promotion intervention
OH: One Health
PAR: Participative Action Research
NABUCO: Nature-based burnout coaching intervention
NBI: Nature-based intervention
NBC: Nature-based coaching
NBT: Nature-based training
NC: Nature connectedness
BDT: Biophilic Design team.

## References

[1] World Health O. 11th Revision of the International Classification of Diseases (ICD-11), Inclusion of Burnout as an Occupational Phenomenon. Available online at: https://www.who.int/news/item/28--05-2019-burn-out-an-occupational-phenomenon-international-classification-of-diseases (accessed, May 1, 2021).

[2] Chabot P. Filosofie van de Burnout. Amsterdam: Amsterdam University Press (2018). 137p.

[3] Golembiewski RT Boudreau RA Munzenrider RF Luo H. Global Burnout: A Worldwide Pandemic Explored by the Phase Model. Amsterdam: Jai Press Greenwich, England (1996).

[4] Schaufeli WB. Burnout in Europe: Relations With National Economy, Governance, and Culture. Research Unit Occupational & Organizational Psychology and Professional Learning (internal report) KU Leuven, Belgium (2018).

[5] Jha AK Iliff AR Chaoui AA Defossez S Bombaugh MC Miller YR. A Crisis in Health Care: A Call to Action on Physician Burnout. Waltham, MA: Massachusetts Medical Society, MHaHA, Harvard TH Chan School of Public Health, and Harvard Global Health Institute (2019). Available online at: https://www.massmed.org/Publications/Research,-Studies,-and-Reports/Physician-Burnout-Report-2018/ (accessed, March 12, 2021).

[6] Dyrbye LN Shanafelt TD Sinsky CA Cipriano PF Bhatt J Ommaya A. Burnout Among Health Care Professionals: A Call to Explore and Address This Underrecognized Threat to Safe, High-Quality Care. NAM Perspect. (2017). 10.31478/201707b

[7] Lemaire JB Wallace JE. Burnout among doctors. BMJ. (2017) 358:j3360. 10.1136/bmj.j3360 28710272

[8] van Mol MMC Kompanje EJO Benoit DD Bakker J Nijkamp MD. The prevalence of compassion fatigue and burnout among healthcare professionals in intensive care units: a systematic review. PLoS ONE. (2015) 10:e0136955. 10.1371/journal.pone.0136955 26322644 PMC4554995

[9] Huberman AM Vandenberghe R. Understanding and Preventing Teacher Burnout: A Sourcebook of International Research and Practice. Cambridge: Cambridge University press (1999). 10.1017/CBO9780511527784

[10] Golembiewski RT Boudreau RA Sun B-C Luo H. Estimates of burnout in public agencies: worldwide, how many employees have which degrees of burnout, and with what consequences? Public Adm Rev. (1998) 58:59–65. 10.2307/976890

[11] Schaufeli Wilmar B Leiter Michael P Maslach C. Burnout: 35 years of research and practice. Career Dev Int. (2009) 14:204–20. 10.1108/13620430910966406

[12] Maslach C Schaufeli WB Leiter MP. Job burnout. Ann Rev Psychol. (2001) 52:397–422. 10.1146/annurev.psych.52.1.397 11148311

[13] Desart S Schaufeli W De Witte H. Op zoek naar een nieuwe definitie van burn-out. Tijdsch Steunpunt Werk. (2017) 1:90–1. Available online at: https://kuleuvenblogt.be/2017/02/20/op-zoek-naar-een-nieuwe-definitie-van-burn-out (accessed January 22, 2021).

[14] Schaufeli W De Witte H Desart S. Manual Burnout Assessment Tool (BAT). Leuven: KU (2019). 10.3390/ijerph17249495 PMC7766078 33352940

[15] Zimmerman BJ Dubois N Houle J Lloyd S Mercier C Brousselle A. How does complexity impact evaluation: an introduction to the special issue. Can J Program Eval. (2011) 26:5–20. 27274614 PMC4891190

[16] Mikolajczak M Gross JJ Stinglhamber F Lindahl Norberg A Roskam I. Is parental burnout distinct from job burnout and depressive symptoms? Clin Psychol Sci. (2020) 8:673–89. 10.1177/2167702620917447

[17] Bianchi R Truchot D Laurent E Brisson R Schonfeld IS. Is burnout solely job-related? A critical comment. Scand J Psychol. (2014) 55:357–61. 10.1111/sjop.12119 24749783

[18] Awa WL Plaumann M Walter U. Burnout prevention: a review of intervention programs. Patient Educ Couns. (2010) 78:184–90. 10.1016/j.pec.2009.04.008 19467822

[19] Maslach C Goldberg J. Prevention of burnout: new perspectives. Appl Prev Psychol. (1998) 7:63–74. 10.1016/S0962-1849(98)80022-X

[20] Maslach C. Finding solutions to the problem of burnout. Consult Psychol J. (2017) 69:143–52. 10.1037/cpb0000090

[21] Keune H Flandroy L Thys S De Regge N Mori M van den Berg T. European Onehealth/Ecohealth Workshop Report. Brussels: Belgian Community of Practice Biodiversity and Health, Belgian Biodiversity Platform (2017). 10.1186/s13690-017-0232-6

[22] Rüegg SR Häsler B Zinsstag J. Integrated Approaches to Health: A Handbook for the Evaluation of One Health. Wageningen: Wageningen Academic Publishers (2018). 256 p. 10.3920/978-90-8686-875-9

[23] Forget G Lebel J. An ecosystem approach to human health. Int J Occupat Environ Health. (2001) 7:2(Suppl S3–38). 10.1590/s0102-311x2001000700015 11387989

[24] Bauer G Davies JK Pelikan J. Euhpid Theory Working Group and The Euhpid Consortium. “The EUHPID Health Development Model for the classification of public health indicators.” Health Promotion Int. (2006) 21:153–9. 10.1093/heapro/dak002 16401640

[25] Barton H Grant M. A health map for the local human habitat. J R Soc Promot Health. (2006) 126:252–3. 10.1177/1466424006070466 17152313

[26] Allen J Balfour R Bell R Marmot M. Social determinants of mental health. Int Rev Psychiatry. (2014) 26:392–407. 10.3109/09540261.2014.928270 25137105

[27] Halbesleben JRB Buckley MR. Burnout in organizational life. J Manag. (2004) 30:859–79. 10.1016/j.jm.2004.06.004

[28] Stokols D Hall KL Taylor BK Moser RP. The science of team science: overview of the field and introduction to the supplement. Am J Prev Med. (2008) 35:S77–89. 10.1016/j.amepre.2008.05.002 18619407

[29] Tromp C. Wicked Philosophy. Amsterdam: Amsterdam University Press (2018). 204 p. 10.1017/9789048541096

[30] Dallimer M Irvine KN Skinner AM Davies ZG Rouquette JR Maltby LL. Biodiversity and the feel-good factor: understanding associations between self-reported human well-being and species richness. BioScience. (2012) 62:47–55. 10.1525/bio.2012.62.1.9

[31] Kaplan S. The restorative benefits of nature: toward an integrative framework. J Environ Psychol. (1995) 15:169–82. 10.1016/0272-4944(95)90001-2

[32] Fuller RA Irvine KN Devine-Wright P Warren PH Gaston KJ. Psychological benefits of greenspace increase with biodiversity. Biol Lett. (2007) 3:390–4. 10.1098/rsbl.2007.0149 17504734 PMC2390667

[33] Barton J Pretty J. What is the best dose of nature and green exercise for improving mental health? A multi-study analysis. Environ Sci Technol. (2010) 44:3947–55. 10.1021/es903183r 20337470

[34] Martin L White MP Hunt A Richardson M Pahl S Burt J. Nature contact, nature connectedness and associations with health, wellbeing and pro-environmental behaviours. J Environ Psychol. (2020) 68:101389. 10.1016/j.jenvp.2020.101389

[35] Mayer FS Frantz CM Bruehlman-Senecal E Dolliver K. Why is nature beneficial? The role of connectedness to nature. Environ Behav. (2009) 41:607–43. 10.1177/0013916508319745

[36] Hartig T Mang M Evans GW. Restorative effects of natural environment experiences. Environ Behav. (1991) 23:3–26. 10.1177/0013916591231001

[37] Hartig T van den Berg AE Hagerhall CM Tomalak M Bauer N Hansmann R. Health benefits of nature experience: psychological, social and cultural processes. In: Nilsson K Sangster M Gallis C Hartig T de Vries S Seeland K editors. Forests, Trees and Human Health. Dordrecht: Springer Netherlands (2011). p. 127–68. 10.1007/978-90-481-9806-1_5

[38] Pretty J Peacock J Sellens M Griffin M. The mental and physical health outcomes of green exercise. Int J Environ Health Res. (2005) 15:319–37. 10.1080/09603120500155963 16416750

[39] Ulrich RS Simons RF Losito BD Fiorito E Miles MA Zelson M. Stress recovery during exposure to natural and urban environments. J Environ Psychol. (1991) 11:201–30. 10.1016/S0272-4944(05)80184-7

[40] Maas J Verheij RA Groenewegen PP de Vries S Spreeuwenberg P. Green space, urbanity, and health: how strong is the relation? J Epidemiol Community Health. (2006) 60:587–92. 10.1136/jech.2005.043125 16790830 PMC2566234

[41] Hartig T Evans GW Jamner LD Davis DS Gärling T. Tracking restoration in natural and urban field settings. J Environ Psychol. (2003) 23:109–23. 10.1016/S0272-4944(02)00109-3

[42] Bragg R Atkins G. A Review of Nature-Based Interventions for Mental Health Care. Natural England Commissioned Reports (2016). p. 204.

[43] Zelenski JM Dopko RL Capaldi CA. Cooperation is in our nature: nature exposure may promote cooperative and environmentally sustainable behavior. J Environ Psychol. (2015) 42:24–31. 10.1016/j.jenvp.2015.01.005

[44] Frumkin H Bratman GN Breslow SJ Cochran B Kahn PH Jr Lawler JJ. Nature contact and human health: a research agenda. Environ Health Perspect. (2017) 125:075001. 10.1289/EHP1663 28796634 PMC5744722

[45] Buzzell L Chalquist C. Ecotherapy: Healing With Nature in Mind. Catapult (2010).

[46] McGeeney A. With Nature in Mind: The Ecotherapy Manual for Mental Health Professionals. London: Jessica Kingsley Publishers (2016).

[47] Shanahan DF Astell-Burt T Barber EA Brymer E Cox DTC Dean J. Nature-Based interventions for improving health and wellbeing: the purpose, the people and the outcomes. Sports. (2019) 7:141. 10.3390/sports7060141 31185675 PMC6628071

[48] Gritzka S MacIntyre TE Dörfel D Baker-Blanc JL Calogiuri G. The effects of workplace nature-based interventions on the mental health and well-being of employees: a systematic review. Front Psychiatry. (2020) 11:323. 10.3389/fpsyt.2020.00323 32411026 PMC7198870

[49] Sahlin E Ahlborg G Matuszczyk J Grahn P. Nature-Based stress management course for individuals at risk of adverse health effects from work-related stress—effects on stress related symptoms, workability and sick leave. Int J Environ Res Public Health. (2014) 11:6586–11. 10.3390/ijerph110606586 25003175 PMC4078597

[50] Zylstra MJ. Exploring meaningful nature experience connectedness with nature and the revitalization of transformative education for sustainability (dissertation). Stellenbosch University (2014).

[51] Stigsdotter UK Palsdottir AM Burls A Chermaz A Ferrini F Grahn P. Nature-based therapeutic interventions. In: Nilsson K Sangster M Gallis C Hartig T de Vries S Seeland K Schipperijn J editors. Forests, Trees and Human Health. Dordrecht: Springer (2011). p. 309–42. 10.1007/978-90-481-9806-1_11

[52] Jax K Calestani M Chan KMA Eser U Keune H Muraca B. Caring for nature matters: a relational approach for understanding nature's contributions to human well-being. Curr Opin Environ Sustainability. (2018) 35:22–9. 10.1016/j.cosust.2018.10.009

[53] Baxter DE Pelletier LG. Is nature relatedness a basic human psychological need? A critical examination of the extant literature. Can Psychol Psychol Canadienne. (2019) 60:21–34. 10.1037/cap0000145

[54] Nisbet E Zelenski J. The NR-6: a new brief measure of nature relatedness. Front Psychol. (2013) 4:813. 10.3389/fpsyg.2013.00813 24198806 PMC3814587

[55] Lumber R Richardson M Sheffield D. Beyond knowing nature: contact, emotion, compassion, meaning, and beauty are pathways to nature connection. PLoS ONE. (2017) 12:e0177186. 10.1371/journal.pone.0177186 28486515 PMC5423657

[56] Zylstra MJ Knight AT Esler KJ Le Grange LLL. Connectedness as a core conservation concern: an interdisciplinary review of theory and a call for practice. Springer Sci Rev. (2014) 2:119–43. 10.1007/s40362-014-0021-3

[57] Chapin FS III Kofinas GP Folke C Chapin MC. Principles of Ecosystem Stewardship: Resilience-Based Natural Resource Management in a Changing World. Springer Science & Business Media (2009).

[58] Chen-Yen C Ping-Kun C. Human response to window views and indoor plants in the workplace. HortScience HortSci. (2005) 40:1354–9. 10.21273/HORTSCI.40.5.1354

[59] Kaplan R. The role of nature in the context of the workplace. Landsc Urban Plan. (1993) 26:193–201. 10.1016/0169-2046(93)90016-7

[60] Largo-Wight E Chen WW Dodd V Weiler R. Healthy workplaces: the effects of nature contact at work on employee stress and health. Public Health Rep. (2011) 126:124–30. 10.1177/00333549111260S116 21563720 PMC3072911

[61] Thompson A Bruk-Lee V. Naturally! examining nature's role in workplace strain reduction. Occup Health Sci. (2019) 3:23–43. 10.1007/s41542-019-00033-5

[62] Kellert S Calabrese E. The Practice of Biophilic Design (2015). Available online at: www.biophilic-design.com

[63] Gillis K GB. A review of psychological literature on the health and wellbeing benefits of biophilic design. Buildings. (2015) 5:948–63. 10.3390/buildings5030948

[64] Ryan CO Browning WD Clancy JO Andrews SL Kallianpurkar NB. Biophilic design patterns: emerging nature-based parameters for health and well-being in the built environment. Int J Archit Res. (2014) 8:62–76. 10.26687/archnet-ijar.v8i2.436

[65] Obiozo RN Smallwood JJ. The intelligent construction workplace: the exceptional creden-tials of the biophilic design concept of the workplace. In: International Council for Research and Innovation in Building Construction CIB TG59. People in Construction Conference. Port Elizabeth, South Africa: Nelson Mandela Metropolitan University (2014). p. 6–8.

[66] Gray T. ‘Retrofitting Biophilic Design Elements into Office Site Sheds: Does ‘Going Green'Enhance the Well-Being and Productivity of Workers’. In: Almusaed A editor. Landscape Architecture: The Sense of Places, Models and Applications. London: IntechOpen Limited (2017). p. 105–26. 10.5772/intechopen.71890

[67] Lauwers L Bastiaens H Remmen R Keune H. Nature's contributions to human health: a missing link to primary health care? A scoping review of international overview reports and scientific evidence. Front Public Health. (2020) 8:52. 10.3389/fpubh.2020.00052 32257986 PMC7093563

[68] Abma T Banks S Cook T Dias S Madsen W Springett J. Participatory Research for Health and Social Well-Being. Cham: Springer (2019). 10.1007/978-3-319-93191-3

[69] Richards DA Hallberg IR. Complex Interventions in Health: An Overview of Research Methods. London: Routledge (2015). 10.4324/9780203794982

[70] Tambuyzer E Pieters G Van Audenhove C. Patient involvement in mental health care: one size does not fit all. Health Expect. (2014) 17:138–50. 10.1111/j.1369-7625.2011.00743.x 22070468 PMC5060706

[71] Kindon S Pain R Kesby M. Participatory Action Research Approaches and Methods: Connecting People, Participation and Place. London: Routledge (2007). 10.4324/9780203933671

[72] van Droffelaar B Jacobs M. Nature-Based training program fosters authentic leadership. J Leadersh Stud. (2018) 12:7–18. 10.1002/jls.21569

